# Synergistic integration of plasmonic and perovskite nanosurfaces to create a multi-gas sensor for environmental monitoring

**DOI:** 10.1039/d4ra06125j

**Published:** 2024-12-17

**Authors:** Desta Regassa Golja, Megersa Olumana Dinka, Alemayehu Getahun Kumela

**Affiliations:** a Department of Civil Engineering Science, Faculty of Engineering and the Built Environment, University of Johannesburg Johannesburg 2006 South Africa mdinka@uj.ac.za; b Department of Applied Physics, School of Applied Natural Sciences, Adama Science and Technology University Adama Ethiopia regsadd12@gmail.com

## Abstract

The escalating levels of air pollution present a critical challenge, posing significant risks to both public health and environmental sustainability. However, recent gas detection methodologies often have inadequate sensitivity and specificity, failing to accurately identify low concentrations of harmful pollutants in real time. Therefore, in this work a (TiO_2_/ZrO_2_)^*N*/2^/CsAgBr_3_/(TiO_2_/ZrO_2_)^*N*/2^-based one dimensional photonic crystal (1D-PC) gas sensor is proposed for detecting key environmental pollutants, specifically ammonia (NH_3_), methane (CH_4_), carbon disulfide (CS_2_), and chloroform (CHCl_3_). Using the transfer matrix method (TMM) and systematically optimizing critical parameters – including the angle of incidence, dielectric layer composition, thickness of the defect layer, and gas concentration – the computational results reveal a maximum sensitivity of 2170 nm per RIU, figure of merit of 500/RIU, detection accuracy of 0.815, and 0.24 quality factor. These findings underscore the potential of the proposed gas sensor as a robust tool for monitoring environmental concentrations of hazardous compounds.

## Abstract 2

A (TiO_2_/ZrO_2_)^*N*/2^/CsAgBr_3_/(TiO_2_/ZrO_2_)^*N*/2^-based one dimensional photonic crystal gas sensor is proposed for detecting key environmental pollutants, specifically ammonia, methane, carbon disulfide and chloroform.

## Graphical abstract

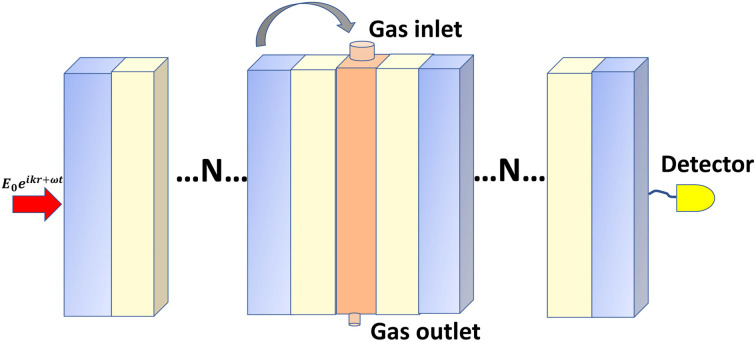

## 1 Introduction

The recent rapid expansion of industrialization, transportation, and urbanization, has resulted in a significant increase in atmospheric pollutant emissions which pose health risks and environmental challenges on a global scale.^1^ Particulate matter, volatile organic compounds (VOCs), nitrogen oxides (NO_*x*_), and sulfur dioxide (SO_2_) are key pollutants contributing to air quality deterioration, leading to respiratory illnesses, cardiovascular issues, and ecological degradation.^2,3^ In response to these growing concerns, various sensing techniques such as mass spectrometry, gas chromatography, and laser gas analysis, have been extensively reported.^4,5^ However, a critical challenge remains in achieving rapid response times, durable performance over multiple cycles, and enhanced selectivity.^6^ As a result, optical sensing technologies like surface plasmon resonance (SPR), fiber optic sensors, and photoluminescence-based sensors have garnered significant attention for their advantages including real-time monitoring, superior sensitivity, and potential for multiplexing capabilities.^7^

Despite the significant advancements in optical gas sensor technologies, the quest for improved sensing performance, selectivity, and reliability in the area of comprehensive air pollution monitoring, persists as a formidable challenge.^8^ This underscores the imperative for innovative sensor platforms capable of overcoming the constraints of existing detection methodologies and providing heightened sensitivity across a broad spectrum of airborne pollutants.^6^ In this context, the unique periodic structure of photonic crystals which contributes to the creation of bandgaps that can slow light propagation and enhance light–matter interactions, distinguishes them as a premier option for optical gas sensors.^9^ Consequently, various theoretical modelling and fabrication endeavours (1D, 2D, and 3D) are currently in progress.^10^ Overall, the simplicity and ease of fabrication makes 1D photonic crystal (PC) the most widely employed for different sensing applications.^11^

Recent advancements have seen extensive reporting on the experimental fabrication and theoretical modelling of 1D photonic crystal (PC) gas sensors.^12^ In terms of experimental techniques, physical vapor deposition (PVD) has been employed to systematically deposit alternating layers of dielectric materials onto a suitable substrate, thus forming the multilayer structure.^13^ This process is complemented by lithographic techniques, such as photolithography and electron beam lithography, which allow for high-precision patterning of nanoscale features within the PC.^14^ Subsequently, a chemical deposition method is utilized to grow a sensitive sensing layer on the surface of the sensor, thereby enhancing its capability for effective gas detection.^15^ From a theoretical modelling perspective, the investigation of 1D PC-based optical sensors incorporates finite-difference time-domain (FDTD) (which provides comprehensive insights into complex geometries),^16^ Finite Element Method (adeptly addresses irregular configurations),^17^ and transfer matrix method (TMM) (excels in computing the reflection and transmission coefficients of multilayer structures by employing matrix representations of the optical properties of each layer),^18^ each offering distinct advantages tailored to specific challenges. Notably, TMM proves particularly beneficial for its efficiency and clarity in elucidating how parameters like the angle of incidence and layer thickness impact gas detection sensitivity and specificity.^10^

For example, Afsari and Sarraf proposed 1D PC with a triangular array of air holes on the silicon-on insulator substrate that consists of a graphene layer deposited on the inner wall of the two cavity air holes for hydrogen sulfide gas sensing. They achieved a sensitivity of 1.2 × 10^4^ nm per RIU and a detection limit of 1.87 × 10^−6^ RIU.^19^ Besides, Ahmed *et al.* theoretically investigated the Tamm plasmon resonance based photonic bandgap comprising a gas cavity sandwiched between a one-dimensional porous silicon photonic crystal and a Ag layer deposited on a prism. They reported the ultra-high sensitivity (*S* = 1.9 × 10^5^ nm per RIU) and a low detection limit (DL = 1.4 × 10^7^ RIU).^12^ While the reported work has shown remarkable performance, it does not consider the humidity, temperature, and gas flow rate. Thus, Jia *et al.* experimentally fabricated a photonic crystals (PhCs) sensor with water retention using dip-coating with poly(methyl methacrylate) (PMMA) colloidal microspheres, followed by embedding in k-carrageenan/polyacrylamide-ethylene glycol (k-CA/PAM-EG) hydrogel for hazardous gas sensing at low temperature. They reported, at room temperature, that the reflection wavelength of the sensor blue-shifted 49 nm in ammonia, and the structural color changed from red to yellow. For low temperatures, the sensor showed great water retention and antifreezing properties even at 57 °C, due to the double network.^20^ Moreover, the introduction of a defect layer (such as functionalized polymer, metal–organic framework, graphene, quantum dots, carbon nanotube and plasmonic layers) into a 1D photonic crystal gas sensor plays a critical role in facilitating selective gas adsorption, enhancing sensitivity, enabling gas recognition, amplifying signals, and ensuring long-term stability of the sensing platform.^21^

On the other hand, the use of perovskite nanosurfaces in gas sensor applications shows significant potential because of their large specific surface area, adjustable bandgap, and excellent charge conduction properties.^22^ These features enable the precise control of sensing capabilities, ensuring the selective detection of specific gases and quick response times.^23^ Despite these advancements, challenges such as sensitivity to environmental changes and potential degradation from exposure to certain gases, present obstacles that call for further investigation and refinement to achieve optimal performance in gas sensing applications.^24^

Therefore, in this work, we conducted a rigorous numerical analysis of 1D PCs using TMM with MATLAB and CST Studio computational tools. By meticulously controlling key design parameters such as angle of incidence, layer thickness, periodicity, and the number of layers, we successfully engineered 1D PCs to exhibit tailored photonic bandgaps (PBGs) for enhanced gas sensing applications. Our investigation further illuminated the influences of critical environmental factors including humidity, gas flow rate, and temperature, on the sensing performance, as evidenced by key metrics such as figure of merit, sensitivity, limit of detection, and quality factors. In addition, we evaluated the sensor's efficacy in detecting multiple toxic gases, affirming its versatile applicability in real-world scenarios. This comprehensive approach not only advances the understanding of photonic crystal structures in gas detection but also contributes essential insight for the development of sophisticated and reliable environmental monitoring technologies.

## 2 Theoretical model and parameters

The interplay between temperature, relative humidity, and pressure is crucial in a gas sensor. Hence, a higher temperature typically holds more water vapor, and as it cools, the relative humidity reaches saturation, leading to dew formation (dew point temperature). The relationship between the air temperature, dew point temperature, and relative humidity indicates the level of water vapor saturation.^25^ With this general fact we propose a photonic crystal gas sensor constituting air/(AB)^*N*/2^/CsAgBr_3_/(AB)^*N*/2^/air. Where A represents a low refractive index layer (SiO_2_, Al_2_O_3_, and ZrO_2_) and B represents the high refractive index metal oxides (TiO_2_ and Si_3_N_4_) utilized in the middle of the perovskite-based cavity.

**Fig. 1:**
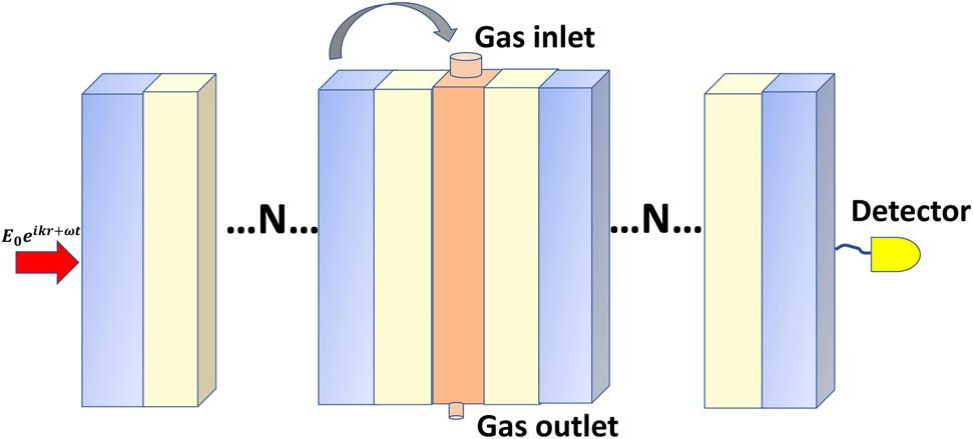
Schematic diagram of the proposed photonic crystal gas sensor that constitutes the periodic arrangement of low refractive index layer and high refractive index layer sandwiched by a perovskite cavity.

In selecting each layer, we consider some optical and chemical properties, for example in selecting Al_2_O_3_ and Si_3_N_4_ provides high transparency in the visible and near-infrared regions, which enables effective light interaction, crucial for sensor functionality.^26,27^ The high refractive index enhances light confinement in multilayer structures, improving sensitivity towards detected gases, and demonstrates strong chemical stability and resistance to environmental degradation, making it an ideal protective layer that supports consistent performance in gas sensing applications.^28,29^ Further, CsAgBr_3_ and CsGeBr_3_ exhibit unique optical properties that make them particularly well-suited for gas sensor applications compared to other halide-based perovskites. Both materials exhibit high photoluminescence efficiency and favorable exciton binding energies, which boost their sensitivity to gas adsorption, facilitating efficient charge generation and transfer. Moreover, the broad PBG and ability to tune optical properties through compositional modifications, enable effective light absorption across a range of wavelengths and therefore enhances their effectiveness in detecting specific gaseous compounds.^30,31^ While CsGeBr_3_ is acclaimed for its remarkable optical and electronic properties, it is inherently susceptible to moisture, which may result in hydrolysis and subsequent structural degradation, thereby necessitating protective coatings to ensure long-term stability.^32^ In addition to this moisture sensitivity, CsGeBr_3_ exhibits thermal instability, with the potential for phase changes occurring at elevated temperatures, and prolonged exposure to ultraviolet (UV) light may lead to photodegradation, introducing defects that can impair its overall performance.^33^ Also, although CsGeBr_3_ exhibits lower susceptibility to certain chemical degradation pathways in comparison to other halide perovskites, exposure to environmental pollutants still poses a risk to its integrity.^34^ To effectively address these challenges, we propose the application of TiO_2_ and ZrO_2_ coatings on the perovskite layer, which confer excellent thermal and chemical stability, optical transparency across a broad spectral range, and robust resistance to corrosion, acids, bases, and organic solvents, thereby making it exceptionally suitable for a variety of environments (Fig. 1).^35^ Furthermore, the mechanical strength and resilience of these coatings under stress are critical for maintaining the integrity of multilayer structures during thermal cycling or mechanical disturbances, ensuring that optimal optical properties and structural integrity are preserved across a wide range of applications, including gas sensors and optical coatings.^36^

The wavelength dependent refractive index of each layer is given in eqn (1a) to (1d),^11,18^1a
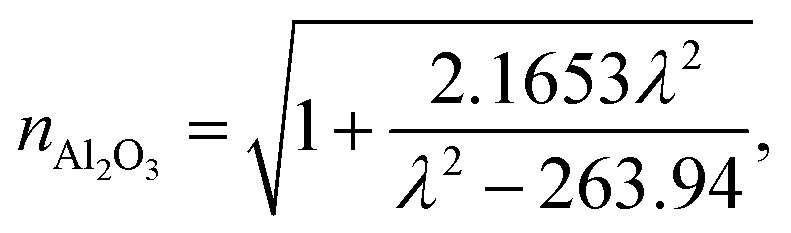
1b
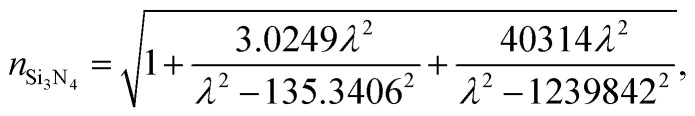
1c
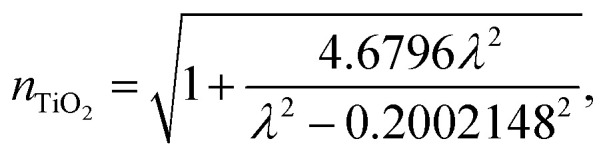
1d
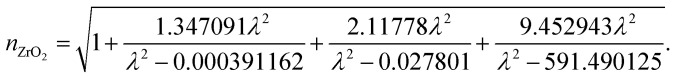


However, the defect layer is treated differently, hence it consists of perovskite cavity and gas molecules interaction. So, employing Bruggeman's effective medium approximation (BEMA), we set the refractive index of the defect layer as,^10^2
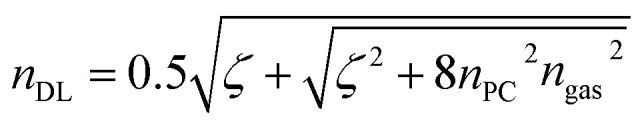
where, *ζ* = 3*P*(*n*_gas_^2^ − *n*_PC_^2^) in which *P* is the porosity ratio that decreases as the perovskite cavity fills with gas, *n*_gas_ is the refractive index of gas, and *n*_PC_ is the refractive index of the perovskite cavity. Finally, the thickness of each dielectric layer is 
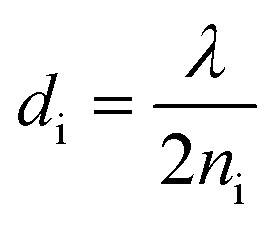
, where i represents each layer.

Once the proposed sensor structure was settled, we proceeded with signal interpretation where the sensor was exposed to an intense electromagnetic field (**E** = **E**_0_ exp(*ikr* – *ωt*)). For an acoustic wave that enters the 1D PC structure from the left side, the governing equations of motion within each layer can be expressed using eqn (3). Thus, the constrictive and destructive interference happens due to the periodic arrangement of RI variation expressed employing TMM as given by,^18^3
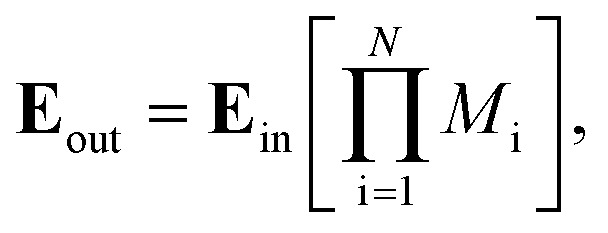
*M*_i_ = (*m*_i_)^*N*/2^*m*_DL_(*m*_i_)^*N*/2^,
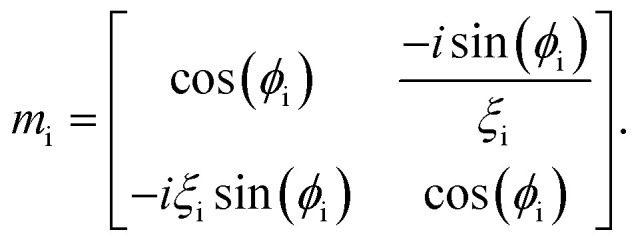
where, *M*_i_ is the characteristic matrix of each layer dispensed in-terms of environmental factor (EF) (humidity, temperature, and gas flow rate), with 
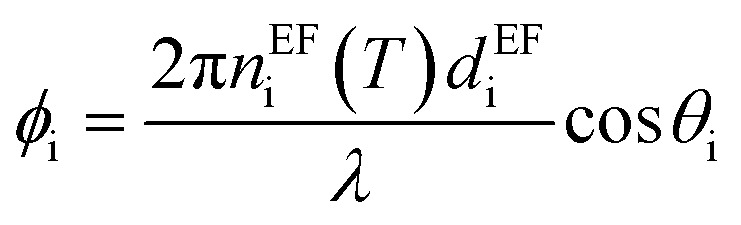
, and 
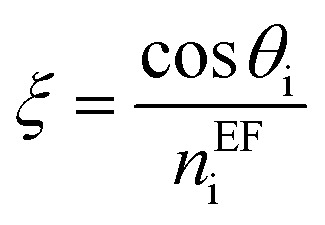
 where *θ* is the angle of incidence by which the electromagnetic field enters into the layer. The change in refractive index and thickness due to EF takes the form,^11^

4 *n*^EF^_i_ = *n*_i_(1 + *β*_i_Δ*T*),

*d*^EF^_i_(*T*) = *d*_i_(1 + *α*_i_Δ*T*).Here, *α*_i_ and *β*_i_ are the thermal expansion coefficient and thermo-optical effect function of the defect layer.

Applying eqn (1a) to eqn (4), the reflection coefficient and reflectivity of the proposed photonic crystal gas sensor are,5
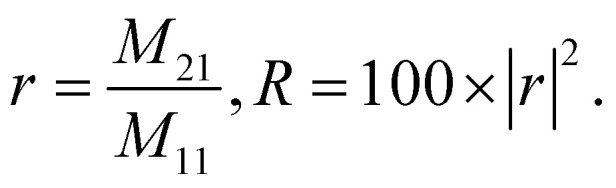


To this end, employing eqn (5), the reflectivity spectra of the proposed gas sensor can be plotted and analyzed using sensitivity (*S*), detection accuracy (DA), quality factor (QF), and figure of merit (FOM), as reported in ref. 7,6
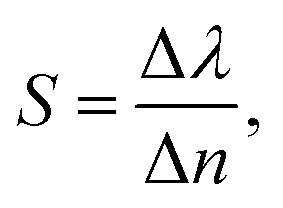

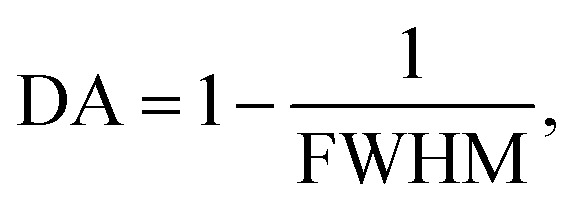

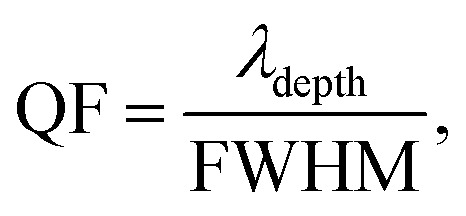

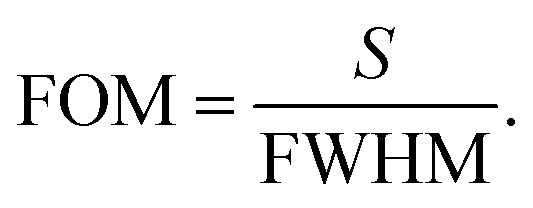


In addition to a clear theoretical model, the following experimental procedures are recommended for practical realization of the proposed 1D PC gas sensor. Initially, the defect layers composed of TiO_2_ and ZrO_2_ are fabricated using pulsed laser deposition (PLD) or laser ablation.^41^ A ceramic target of TiO_2_ and ZrO_2_ is chosen, and a laser beam from a high-energy pulse laser is directed onto the target, causing the material to vaporize and create a plume of atoms and particles.^42^ This plume is deposited onto a clean substrate, such as a silicon wafer or glass, in a controlled environment, allowing for the formation of thin films.^43^ By adjusting the laser fluence, repetition rate, and deposition time, precise control over the thickness and uniformity of the TiO_2_/ZrO_2_ defect layers can be achieved, ensuring that the optical properties are tailored to enhance the overall performance of the gas sensor. This process is repeated for *N*/2 cycles to build up the requisite number of defect layers on either side of the CsAgBr_3_ layer.^44^

Following the establishment of the TiO_2_/ZrO_2_ dielectric layers, the next step involves the deposition of the CsAgBr_3_ cavity layer, which serves as the active gas-sensitive component of the sensor. The fabrication of the CsAgBr_3_ perovskite layer can also utilize laser ablation, where a separate ceramic target composed of CsAgBr_3_ is prepared.^45^ Similar to the previous step, the target is subjected to laser ablation in a vacuum or inert atmosphere to minimize contamination. During this process, the vaporized material is deposited directly onto the TiO_2_/ZrO_2_ layers, creating a uniform perovskite film that traps gas molecules for sensitive detection.^46^ Attention must be paid to the substrate temperature and ambient conditions during the deposition to ensure optimal crystallization and stability of the CsAgBr_3_ layer.^47^ After completing the multilayer structure, the layer can undergo characterization using techniques like X-ray diffraction (XRD) to confirm the crystalline phases, and scanning electron microscopy (SEM) to assess layer morphology and thickness. This comprehensive approach using laser ablation for the fabrication of a multilayer photonic crystal gas sensor presents a feasible pathway to achieve high-performance gas sensing applications with tailored optical properties and enhanced sensitivity.^48^

## 3 Results and discussion

**Fig. 2:**
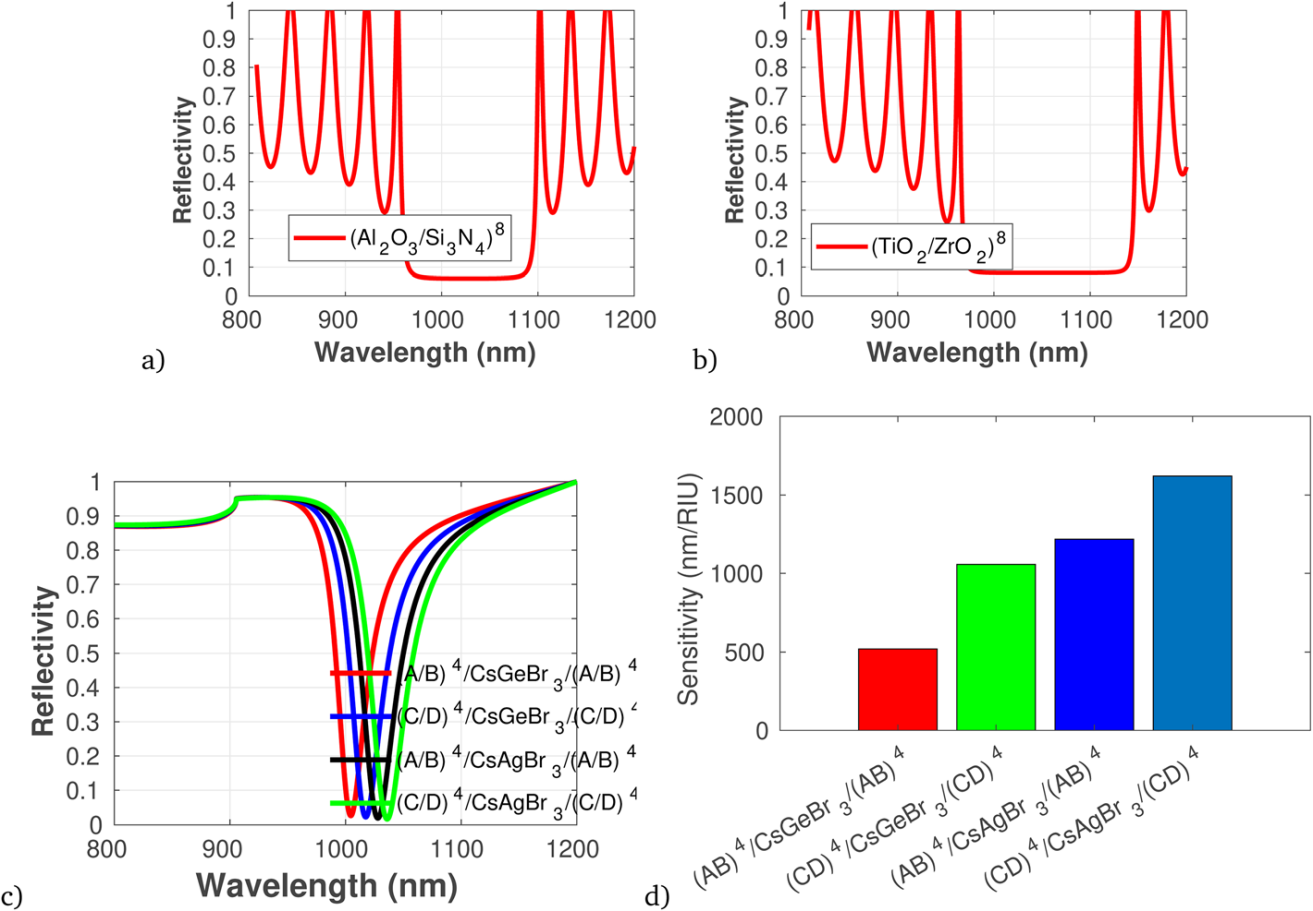
Reflectivity spectra of different dielectric and defect layers of the photonic crystal gas sensor, plotted using the refractive index presented in Table 1 at *θ* = 65*°*, *λ* = 820 nm. (a) and (b) Photonic band structure *versus* wavelength for (Al_2_O_3_/Si_3_N_4_)^8^ and (TiO_2_/ZrO_2_)^8^, respectively. (c) Reflectivity spectra of different dielectric layers and defect layers in which A is Al_2_O_3_, B is Si_3_N_4_, C is TiO_2_, and D is ZrO_2_. (d) Sensitivity in response to defect layer variation.

**Fig. 3:**
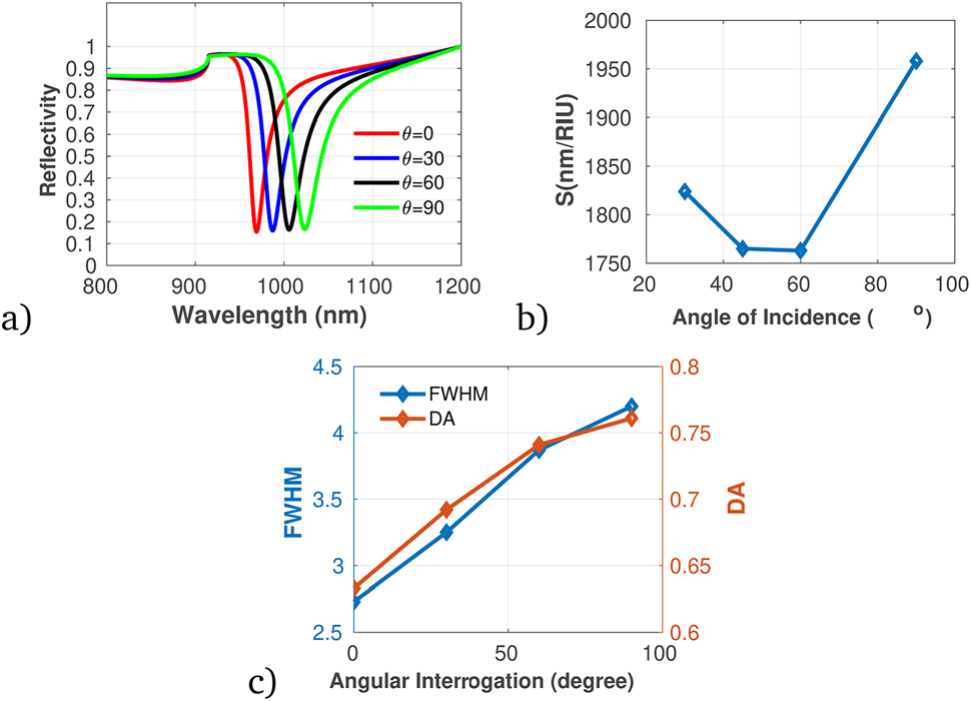
Reflectivity spectra and performance analysis of the proposed 1D-PC gas sensor with varying angle of incidence, plotted using the data presented in Table 1 at *λ* = 820 nm and *N* = 8. (a) Reflectivity *versus* wavelength as a function of angle of incidence. (b) Variation of sensitivity with incidence angle, (c) variation of FWHM and DA with angle of incidence.

**Fig. 4:**
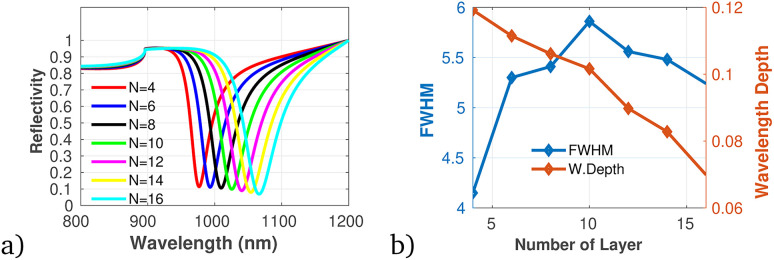
(a) Reflectivity spectra with varying number of dielectric layers. (b) Variation of FWHM and wavelength depth *versus* number of dielectric layers plotted using data presented in Table 1 at *λ* = 820 nm and *θ* = 90°.

**Fig. 5:**
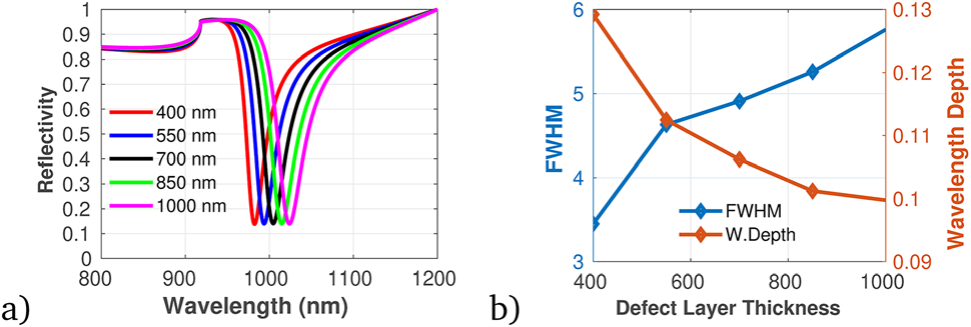
(a) Reflectivity spectra with varying defect layer thickness. (b) Variation of FWHM and wavelength depth *versus* defect layer thickness plotted using data utilized in Table 1 at *λ* = 820 nm, *N* = 10, and *θ* = 90°.

As an initial condition, employing eqn (1a) to (6) in-line with the geometrical parameters, the reflection spectra of different components of sensor layers are plotted in Fig. 2 to 5. The reflectivity *versus* wavelength of the proposed 1D-PC gas sensor composed of (Al_2_O_3_/Si_3_N_4_)^8^ and (TiO_2_/ZrO_2_)^8^ without defect are plotted using TMM and Bloch's theorem. In the wavelength range of concern, two PBGs extend from 980 to 1090 nm and from 990 to 1140 nm, respectively. This clearly reveals that the (TiO_2_/ZrO_2_)^8^ based structure has a broad FWHM, but in sensor scenarios a narrower FWHM is associated with higher sensitivity: because a narrower peak allows for better resolution and distinction between closely spaced signals as previously briefly reported.^49^ Calculating the performance analysis of both configurations we find a maximum DA of 0.997 and QF of 4.6 × 10^−4^ for (Al_2_O_3_/Si_3_N_4_)^8^, and DA of 0.993 and QF of 4.9 × 10^−4^ for (TiO_2_/ZrO_2_)^8^. In addition, we have seen the effect of a defect layer by inserting a perovskite cavity defect layer at the center; it clearly shows that the type of perovskite cavity used influences the reflectivity of the proposed 1D-PC gas sensor (Fig. 2d). Then, employing eqn (6), the sensor performance of each structure was investigated. The results show that the sensor structure of (TiO_2_/ZrO_2_)^4^/CsAgBr_3_/(TiO_2_/ZrO_2_)^4^ has the maximum sensitivity, FOM, DA, and QF. Based on this fact, in the following investigation we fixed the arrangement of the proposed 1D-PC gas sensor to (TiO_2_/ZrO_2_)^*N*/2^/CsAgBr_3_/(TiO_2_/ZrO_2_)^*N*/2^.

To proficiently manage the angle of incidence and optimize sensor performance in practical applications, it is imperative to employ adaptable design strategies that account for the diverse environmental conditions and user interactions that may be encountered. This can be accomplished through the integration of sophisticated mechanical adjustments, such as gimbals or articulating mounts, which facilitate real-time modifications of the sensor's orientation to preserve an optimal operational angle. Furthermore, the incorporation of electronic control systems capable of dynamically adjusting sensing parameters in response to incoming data will significantly enhance the sensor's responsiveness to fluctuations in incident angles. In this context, we establish the optimal incident angle by evaluating reflectivity as a function of wavelength, as depicted in Fig. 3a. The results presented in Fig. 3b and c clearly demonstrate the dependency of sensitivity and detection accuracy (DA) on the angle of incidence. Notably, at an incident angle of 90° the sensor attained a maximum sensitivity of 1958 nm/RIU and a detection accuracy of 0.78, underscoring the critical influence of this parameter on the sensor's performance. Subsequently, we aimed to determine the ideal number of dielectric layers, as plotted in Fig. 4a. Utilizing eqn (5), we examined the relationship between FWHM and the number of dielectric layers, which is illustrated in Fig. 4b. The data indicate that as the number of defect layers is varied from 4 to 10, the FWHM initially increases (suggesting an enhancement in sensitivity and resolution), above 12 dielectric layers, the sensor performance diminishes. Based on this analysis, we determined that configuring the sensor with 10 defect layers provides optimal performance. Next, the effect of defect layer thickness on performance of the proposed 1D-PC gas sensor is clearly shown in Fig. 5(a) and (b). From this result, the thickness of the perovskite defect layer is fixed to 800 nm.

**Fig. 6:**
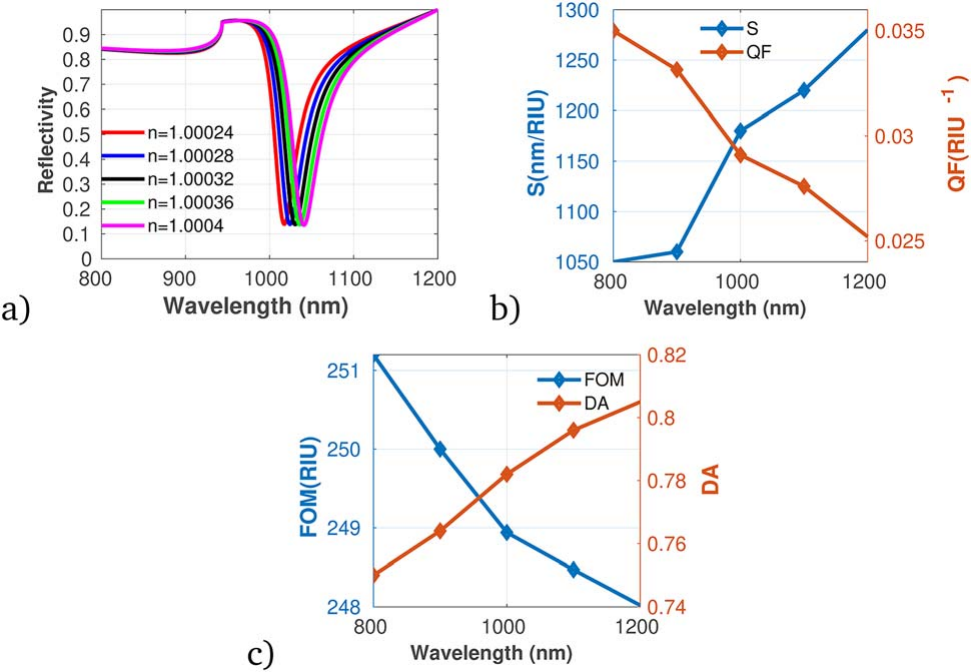
Reflectivity spectra of NH_3_ at varying concentrations plotted utilizing data from Table 1, measured at a wavelength of *λ* = 820 nm, with *N* = 10 layers, an incident angle of (*θ* = 90°) at room temperature. (a) Depicts the variation in reflectivity spectra as a function of the refractive index changes associated with different concentrations of NH_3_. (b) Presents the variations of S and QF with respect to the pumping wavelength. (c) Displays the relationship between the FOM and DA as a function of wavelength.

Utilizing the optimized parameters illustrated in Fig. 2 through 5, the performance analysis of the proposed gas sensor for detecting ammonia (NH_3_) in ambient environments was conducted. The results are presented in Fig. 6a to 6c, which detail the variations in reflectivity, S, QF, DA, and FOM in relation to different concentrations of the pollutant gas. The data indicate that both S and DA exhibit a positive correlation with increasing NH_3_ concentrations, suggesting that the sensor becomes increasingly responsive and capable of accurately identifying NH_3_ as pollutant levels rise. On the other hand, the analysis reveals a decreasing trend in both the QF and the FOM with increasing concentrations of NH_3_, the obtained result directly agrees with the work of Guan *et al.*^50^

**Fig. 7:**
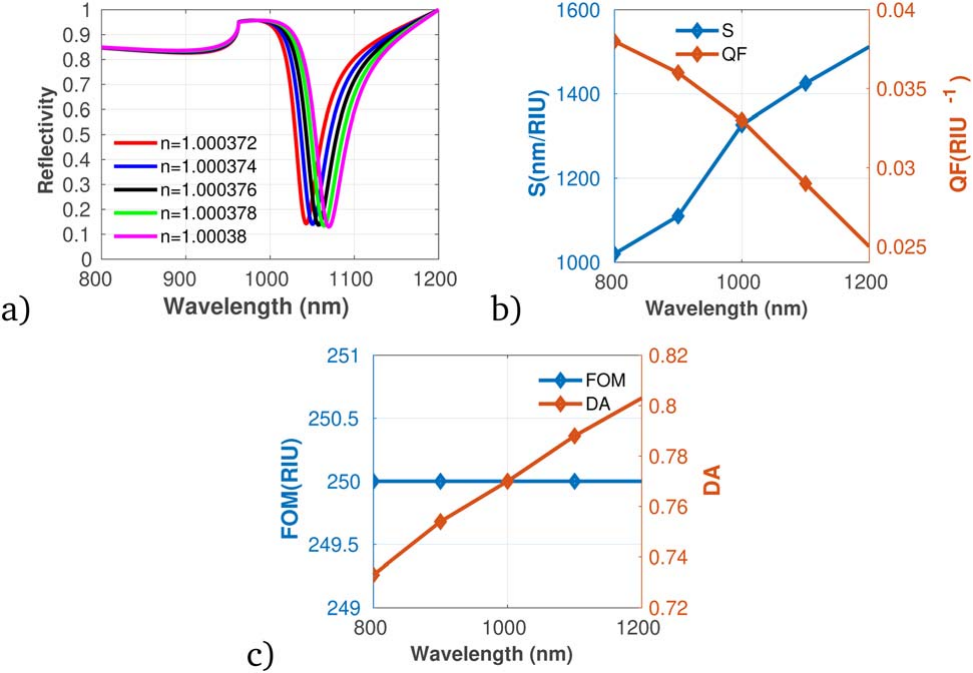
Reflectivity spectra of CH_4_ at varying concentrations plotted utilizing data from Table 1, measured at a wavelength of *λ* = 820 nm, with *N* = 10 layers, an incident angle of (*θ* = 90°) at room temperature. (a) Depicts the variation in reflectivity spectra as a function of the refractive index changes associated with different concentrations of CH_4_. (b) Presents the variations of S and QF with respect to the pumping wavelength. (c) Displays the relationship between the FOM and DA as a function of wavelength.

In addition, the proposed gas sensor was employed for methane (CH_4_) gas detection as shown in Fig. 7a to c. The analysis reveals that both S and DA exhibit a positive correlation with increasing concentrations of CH_4_. While, the QF shows a decreasing trend with rising methane concentrations, indicating that while the sensor becomes more responsive, its precision might diminish at elevated levels of gas exposure. Moreover, the FOM remains constant across the concentration range, reflecting stability in the sensor's overall performance despite changes in concentration. This stability suggests that the sensor can maintain reliable measurements even as it adapts to varying environmental conditions.

**Fig. 8:**
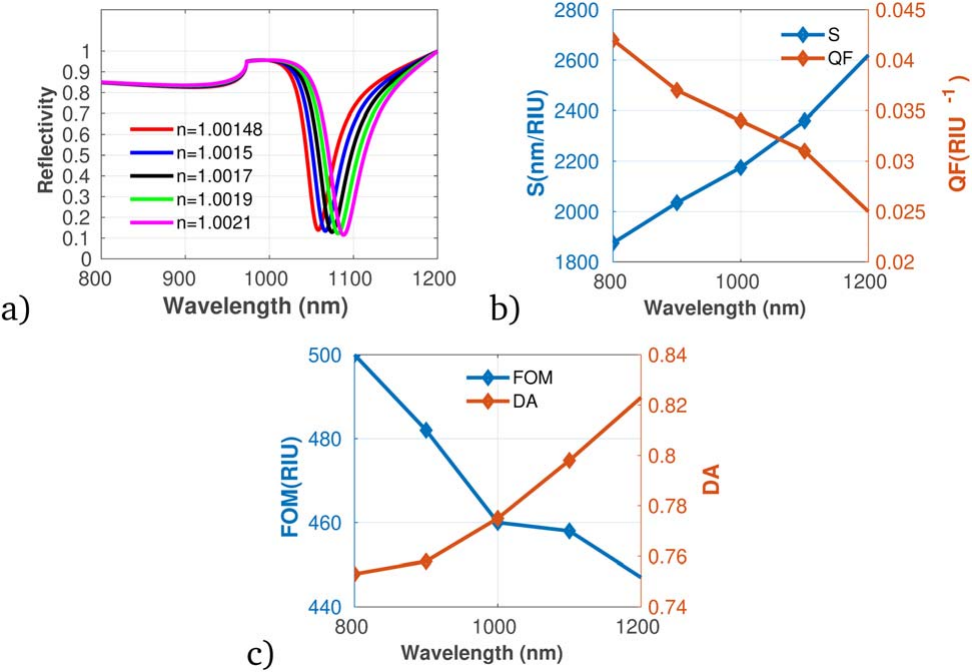
Reflectivity spectra of CS_2_ at varying concentrations plotted utilizing data from Table 1, measured at a wavelength of *λ* = 820 nm, with *N* = 10 layers, an incident angle of (*θ* = 90°) at room temperature. (a) Depicts the variation in reflectivity spectra as a function of the refractive index changes associated with different concentrations of CS_2_. (b) Presents the variations of S and QF with respect to the pumping wavelength. (c) Displays the relationship between the FOM and DA as a function of wavelength.

Fig. 8a to c show the performance of the proposed gas sensor for detecting carbon disulfide (CS_2_). The results indicate that as the concentration of the pollutant gas increases, both S and DA exhibit an upward trend. This enhancement in sensor performance suggests capability for accurately identifying and quantifying CS_2_ at elevated concentrations. Conversely, our analysis reveals a decline in both the QF and FOM with increasing CS_2_ levels. This indicates that while the sensor becomes more responsive to higher CS_2_ concentrations, there may be trade-offs in precision and overall effectiveness, as denoted by the decreasing QF and FOM values. Despite these diminishing metrics, the results affirm that the proposed gas sensor maintains accurate measurements, particularly at lower concentrations of CS_2_. This accuracy is crucial, considering the potential health hazards and environmental impacts associated with CS_2_ emissions.

**Fig. 9:**
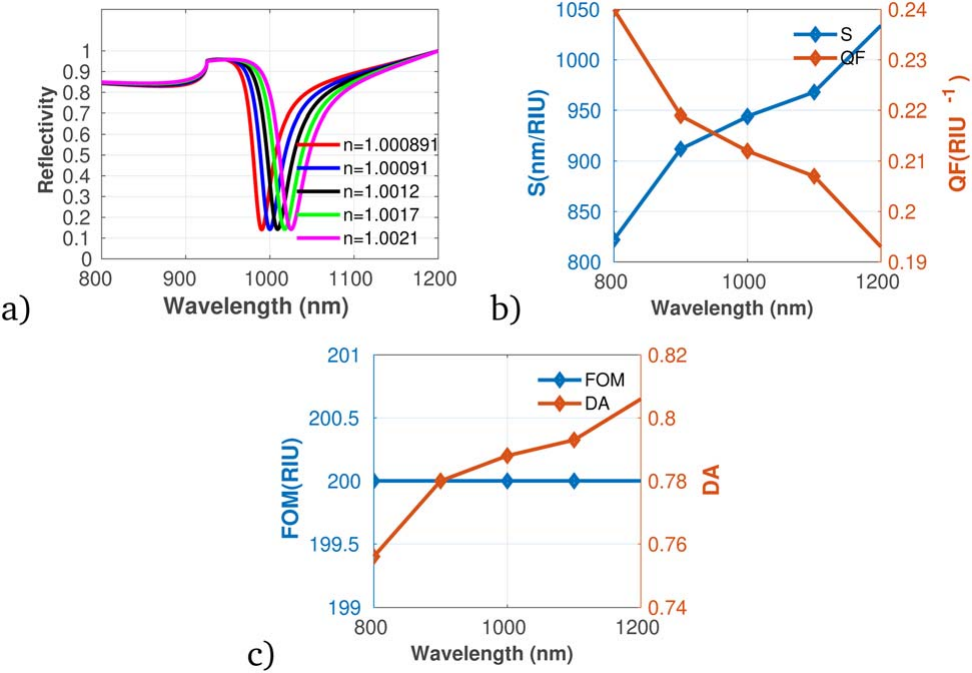
Reflectivity spectra of CHCl_3_ at varying concentrations plotted utilizing data from Table 1, measured at a wavelength of *λ* = 820 nm, with *N* = 10 layers, an incident angle of (*θ* = 90°) at room temperature. (a) Depicts the variation in reflectivity spectra as a function of the refractive index changes associated with different concentrations of CS_2_. (b) Presents the variations of S and QF with respect to the pumping wavelength. (c) Displays the relationship between the FOM and DA as a function of wavelength.

Further, we considered the performance of the proposed gas sensor for the detection of chloroform (CHCl_3_) in the environment (see Fig. 9a to c). The data indicate a significant increase in both S and DA as the concentration of chloroform rises, suggesting that the sensor is effectively calibrated to respond to varying levels of this VOC. Fig. 9b shows a decrease in the QF as the concentration of chloroform increases, which may indicate that while the sensor's responsiveness improves, its precision could be compromised under elevated concentration scenarios. Importantly, the FOM remains stable across the concentration range, suggesting that the overall performance of the sensor does not diminish despite variations in gas levels, which agrees with the result obtained utilizing graphene-metal oxide hybrids.^51^ This is particularly relevant as it indicates the sensor's capability to maintain reliable measurements, especially at low concentrations of chloroform, despite possible fluctuations in other performance metrics.

**Fig. 10:**
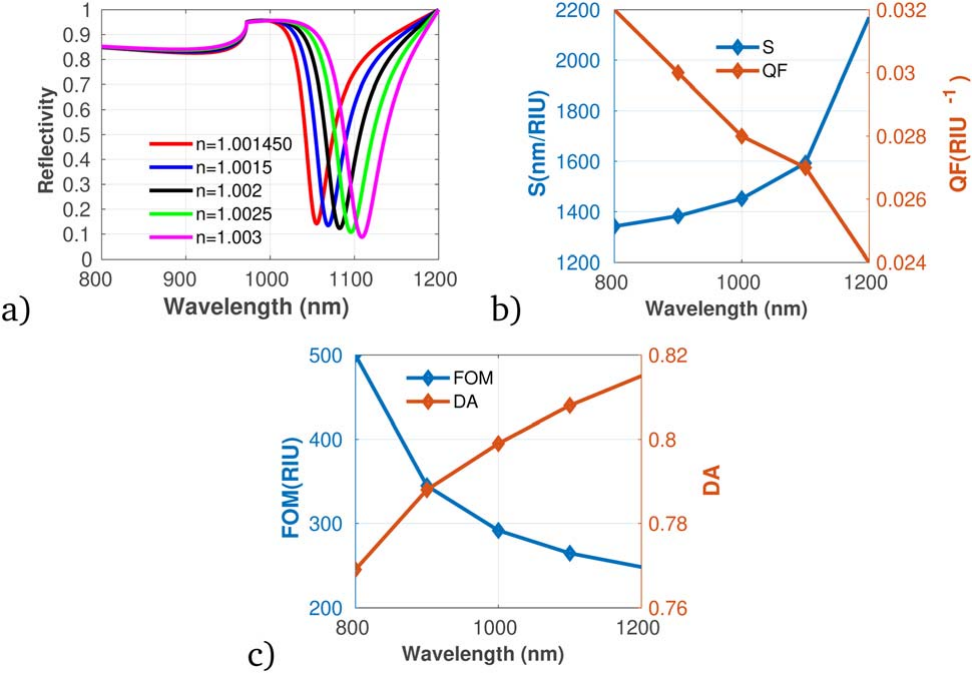
Reflectivity spectra of methyl at varying concentrations plotted utilizing data from Table 1, measured at a wavelength of *λ* = 820 nm, with *N* = 10 layers, an incident angle of (*θ* = 90°) at room temperature. (a) Depicts the variation in reflectivity spectra as a function of the refractive index changes associated with different concentrations of methyl. (b) Presents the variations of *S* and QF with respect to the pumping wavelength. (c) Displays the relationship between the FOM and DA as a function of wavelength.

Finally, we performed a comprehensive performance analysis of the proposed gas sensor for detecting methyl compounds in the environment, as summarized in Fig. 10a to c. The findings indicate a notable increase in both *S* and DA as the concentration of methyl compounds rises, demonstrating the sensor's capability to effectively respond to varying levels of this pollutant. While both the QF and FOM exhibit a substantial decrease with rising concentrations of methyl. Even with these decreasing metrics, the stability of the sensor's overall performance underscores its efficacy, particularly at low concentrations of methyl. The obtained result is agrees with related work based on a composite material of halide metal perovskite, reported by ref. 52.

The selectivity of the sensor in a mixed-gas environment is paramount for achieving accurate detection and measurements of individual pollutants. The proposed gas sensor leverages a sophisticated mechanism that capitalizes on variations in its reflective index, which are intrinsically linked to the concentration and nature of the gas present in the environment. The sensor's response is characterized by shifts in the reflected wavelength spectrum, facilitating the precise identification of multiple gases even in a mixture. This capability arises from the distinct interaction characteristics exhibited by each gas with the photonic crystal structure, which engenders identifiable optical signatures that the sensor can differentiate. As illustrated in Table 2, which summarizes the findings from Fig. 6–10, each pollutant yields a unique wavelength response, effectively establishing distinct spectral “fingerprints” for detection. Importantly, our results demonstrate that the response wavelengths corresponding to varying concentrations of each pollutant do not overlap, ensuring unequivocal differentiation between gases. This characteristic not only highlights the sensor's ability to independently identify each pollutant but also reinforces its practical utility in environmental monitoring scenarios where diverse pollutants coexist. Consequently, the proposed gas sensor holds substantial promise for the accurate detection and comprehensive analysis of multiple pollutants in real-world atmospheric conditions.

**Table 1:** Concentration dependent refraction of different pollutant gases

Gas	Concentration	RI	Ref.
NH_3_	15	1.00024	
	30	1.00028	
	45	1.00032	37
	60	1.00036	

CH_4_	15	1.000372	
	30	1.000374	
	45	1.000376	38
	60	1.000378	

CHCl_3_	15	1.001450	
	30	1.00150	
	45	1.001550	39
	60	1.001560	

CS_2_	15	1.0015	
	30	1.0017	
	45	1.0019	40
	60	1.00121	

**Table 2:** Response of proposed photonic crystal gas sensor under different gas concentrations

Pollutant gas	Concentration	RI	Response wavelength (nm)
NH_3_	15	1.00024	1023
	30	1.00028	1029
	45	1.00032	1033
	60	1.00036	1037

NH_3_	15	1.000372	1040
	30	1.000374	1049
	45	1.000376	1056
	60	1.000378	1062

CHCl_3_	15	1.001450	1050
	30	1.00150	1072
	45	1.001550	1093
	60	1.001560	1050

CS_2_	15	1.0015	1072
	30	1.0017	1093
	45	1.0019	1106
	60	1.00121	1115

Finally, the comparative analysis of the proposed gas sensor with respect to recently reported experimental and theoretical work is displayed in Table 3. It clearly shows that the proposed sensor can be used as a multi-gas sensor with high efficiency.

**Table 3:** Comparison of maximum performance of recently reported optical gas sensors

Sensor configuration	Pollutant gas	*S* (nm per RIU)	FOM (RIU^−1^)	DA	QF	Ref.
Silicon planar hole with defect layer	0_2_, CH_4_, NH_3_	270	—	10^−^4	—	53
MICMI	Phthalate & bisphenol A	2350.51	543	—	0.789	54
MI plasmonic waveguide	CO_2_	1000	133	—	—	55
Silicon nanocavity	N_2_, He and CO_2_	400	258.333	0.65	—	56
MoS2/Au hybrid plasmon-exciton	Volatile organic	1.21	—	—	0.99	57
Ag/MgO/Si	Toxic gas	86.12	5.86	0.17	—	58
GaAs rod PC	Toxic gas	865.2	160	0.988	0.988	59
Micro ring resonator PC	CH_4_	1136	360	—	0.262	60
Si-based plasmonic cavity	CH_2_O & N_2_O	600	353	—	—	61
Ag/Nitride material/WTe2/BP	Toxic gas	249.25	47.38	1.94	0.49	62
(TiO_2_/ZrO_2_)^*N*/2^/CsAgBr_3_/(TiO_2_/ZrO_2_)^*N*/2^	NH_3_, CH_4_, CS_2_ & CHCl_3_	2170	500	0.815	0.733	This work

## 4 Conclusion

In conclusion, this study meticulously investigates the efficacy of a novel gas sensor engineered for the detection of volatile organic compounds, emphasizing its performance across a spectrum of gas types and concentrations. The findings reveal substantial enhancement in both sensitivity (*S*) and detection ability (DA) in correlation with increasing gas concentrations, highlighting the sensor's precision in responding to variations in pollutant levels. Notably, the observed decrease in the quality factor (QF) and the figure of merit (FOM) with rising concentrations, indicate the sensor's capability to maintain accurate measurements, especially at low concentration levels, an essential factor for effective environmental monitoring. The computational analysis demonstrates a maximum sensitivity of 2170 nm/RIU, a FOM of 500/RIU, and a DA of 0.815, along with supplementary performance metrics that collectively affirm the sensor's reliability. These results validate the proposed gas sensor as a robust instrument for real-time assessments of multi-gas pollution, delivering significant implications for the advancement of air quality monitoring and regulatory compliance in environmental management. Consequently, this research makes a significant contribution to the domain of gas detection technology, laying the groundwork for further innovations in sustainable monitoring solutions that are adaptable to the ever-evolving demands of environmental protection and public health safety. Moreover, from an economic perspective, the integration of our advanced sensor technology into large-scale environmental monitoring systems offers considerable advantages over conventional gas detection methods.

## Data availability

Data underlying the results presented in this paper are not publicly available at this time but may be obtained from the authors upon reasonable request.

## Conflicts of interest

The authors declare that they have no known competing financial interests or personal relationships that could have appeared to influence the work reported in this paper.
